# How digital infrastructure supports emerging forms of urban digital tourism in China

**DOI:** 10.1371/journal.pone.0343903

**Published:** 2026-03-12

**Authors:** Jielong Huang, Wenbin Ling, Shanni Ye, Rabnawaz Khan, Qifu Lai

**Affiliations:** 1 School of Internet Business and Economics, Fujian University of Technology, Fuzhou, Fujian, China; 2 School of Economics and Management, Fujian Agriculture and Forestry University, Fuzhou, Fujian, China; Sun Yat-Sen University, CHINA

## Abstract

China’s ongoing digital transformation, supported by proactive government policies, is fundamentally reshaping urban tourism through strategic development of digital infrastructure. This study employs a Difference-in-Differences (DID) model, taking the Broadband China policy as a quasi-natural experiment, to examine the driving effects and mechanisms of digital infrastructure development on emerging forms of urban digital tourism entrepreneurship. The results show that: (1) digital infrastructure development can facilitate the development of emerging forms for urban digital tourism entrepreneurship. Even after controlling for endogeneity, other policy intervention, and robustness in parallel trend testing, the outcome remains unchanged. Digital infrastructure has the potential to foster the growth of new types of urban digital tourism businesses by increasing the concentration of digital talent, the rate of technological innovation and application, and the level of government support. The driving effect is stronger in cities that have exceptional cultural value, well-allocated administrative resources, and excellent tourist resources. Coordination and information exchange in industrial chains are beneficial for nearby regions, but they pose a threat of marginalization to areas that aren’t immediately adjacent due to the impacts of resource siphoning. Therefore, the government should promote the transformation of urban tourism into intelligent and convenient, establish cross-city tourism alliances, and form a complementary mode of “technology and resources.” Simultaneously, it should make use of digital technology to accurately match the characteristics of the city and guide operators of the industry to efficiently make use of digital technology to identify market opportunities, integrate resources, and unleash the new kinetic energy of the industry’s development.

## 1. Introduction

The new industry is an important engine for the tourism industry to move towards high-quality development. Interactions between digital and traditional industries have always led to new ways of industrial development, new types of industry evolution, and new dynamics of economic growth, all because the digital economy is growing at a rapid pace [1,2]. Owing to the rapid application and iteration of digital devices and technologies such as digital infrastructure, digital communication level, digital operation platform, and big data analysis, data and digital technologies have been widely developed and used as new tourism resources in the tourism industry, which has led to the continuous generalization of the concept of traditional tourism resources and the blurring of tourism development models. Creating more new development situations, giving rise to a large number of emerging forms of digital tourism such as “virtual tourism, metaverse tours, digital tourism, and intelligent tourism,” and continuously driving the innovation and integration of tourism forms [3,4] are all examples of the various ways in which digital tourism is transforming the tourism industry. The size of the market for digital tourism in China increased from 442.77 billion yuan to 969.81 billion yuan between the years 2015 and 2022, as indicated by the data collected for monitoring purposes. The scale of the market reached 1,150 billion yuan by the end of 2023, representing a year-on-year increase of 18.58%, and the culture tourism industry’s share of the market exceeded 30 percent. From the connotation perspective, the new industry of digital tourism is a new model of tourism development, operation, and management formed by taking the data elements and digital technology as the core driving force and by deeply integrating industrialized digital products and digital services with the traditional tourism industry to satisfy tourists’ growing demand for a better tourism experience and meet the requirements of the innovative development of the tourism industry in the era of the digital economy [5]. The growth of digital tourism has garnered a lot of attention and acclaim because it is a key manifestation of emerging kinds of tourism. Destinations all over the world are actively encouraging leapfrog development in tourist innovation in order to capitalize on the powerful driving force and leadership of the digital economy. In this way, it is possible to enable the innovative application of regional tourism resources, optimize the reorganization of the tourism industry, and continuously innovate tourism development models, which makes it an essential driver of economic growth.

Due to the uneven distribution of tourism resources and historical path dependence, China’s tourism industry has spatially concentrated in prefecture-level cities for decades. These urban destinations remain the core attractions for most tourists [6]. To illustrate the preeminent role of urban tourism, in 2023, 333 million tourists visited Wuhan in Hubei Province, which accounted for 44.46% of provincial tourist arrivals and 53.85% of tourism income. Natural scenery, historic sites, and the homes of famous people continue to be the main draws for the majority of cities [7,8]. There has been a marked standardization of products in China’s urban tourism sector as a result of this resource-dependent development paradigm.

With evolving tourist demands, traditional urban tourism faces declining attractiveness and sustainability challenges [9]. Consequently, digital transformation has emerged as a crucial pathway for revitalizing traditional tourism resources and achieving innovative urban tourism development. Weak digital infrastructure, high transformation costs, path dependency, and an absence of market-driven structural modifications are some of the challenges that digital technology integration faces. Digital infrastructure’s part in urban innovation has been well-studied in the scholarly literature. According to research, digital infrastructure boosts entrepreneurial vitality by lowering factor input prices [10], improving governance [11], and reducing operational uncertainties [2]. Urban entrepreneurship stimulates regional economic development by facilitating knowledge diffusion and breakthrough innovations [12]. While these studies highlight the broad economic impacts of digital infrastructure, they lack industry-specific analyses, particularly regarding tourism sector innovation.

Existing tourism research also exhibits limitations. Although scholars recognize industrial integration [13] and digital technologies [8] as catalysts for emerging tourism forms, while there have been some research on topics like virtual tourism and immersive experiences, digital tourism entrepreneurship has received less attention. There is a lack of empirical evaluations of the effects of particular digital policies, despite the widespread agreement that government policies are essential for directing healthy sector development [14]. There is an immediate need to assess the efficacy of policies aimed at enhancing urban tourism structures and encouraging sustainable development models, and this void stands out in light of the widespread adoption of digital technologies.

This study makes three marginal contributions. First, it focuses on the concept of digital tourism, empirically verifies the impact of digital infrastructure on emerging forms of urban digital tourism entrepreneurship, and explores the spatial spillover effect, which enriches the relevant research results. Second, this study verified the intrinsic mechanism by which digital infrastructure affects the development of emerging forms of urban digital tourism and deepened our understanding of the theoretical logical relationship between digital infrastructure, entrepreneurial costs, and emerging forms of urban digital tourism. Third, it confirms the heterogeneous influence of the three factors of resource endowment, administrative authority, and cultural value; points out the direction of future development; and offers substantial opportunities for the relevant parties to further improve the digital infrastructure and promote the innovative development of emerging forms of urban digital tourism.

## 2. Policy background and theoretical analysis

### 2.1 Policy background

Digital tourism, which has emerged at the intersection of digital technologies, the digital economy, and traditional tourism, has become a strategic priority in China’s policy agenda. The Ministry of Culture and Tourism’s Opinions on Deepening “Internet + Tourism” and Promoting High-Quality Tourism Development (2020) explicitly links digital infrastructure advancement to industrial upgrading, advocating enhanced policy-platform-technology synergies to strengthen the digital cultural sector. The 14th Five-Year Plan for Cultural and Tourism Technological Innovation (2021) further codified this course of action by placing an emphasis on smart tourism development via innovations in digital transformation, the integration of the “Internet + Tourism” concept, and new models of cultural-tourism consumption. As part of its ongoing endeavor to adapt policy frameworks to the changing digital landscape, the ministry has been selecting top digital innovation demonstration cases in culture and tourism every year since 2022. These cases exhibit innovative solutions to pressing problems in the field. While these initiatives do shed light on China’s approach to digital tourism development, there are still many unanswered questions, such as how to optimize digital infrastructure, how to tailor tactics to local resources, and how to foster the systemic expansion of digital tourism.

China’s digital infrastructure advancement has been significantly propelled by landmark policies, such as the State Council’s Broadband China Strategy and Implementation Plan (2013), a cornerstone initiative for enhancing global competitiveness in the digital economy. The initiative established a framework for regional specialization in digital infrastructure development through three annual selections of pilot cities (2014–2016) that were accompanied by substantial policy and financial support. Key implementation measures included coordinated regional broadband development with differentiated strategies; infrastructure optimization through innovative public-private partnerships; enterprise digital transformation promotion; industry chain enhancement via collaborative innovation; and cybersecurity capacity building.

Notably, the development of innovative digital tourism forms serves as a critical metric for evaluating the strategy’s effectiveness in fostering digital entrepreneurship and industry transformation. Pilot cities’ experiences provide helpful information about leveraging localized digital advantages while addressing persistent challenges in cross-sectoral resource integration and path-dependent development models.

### 2.2 Core concept definition

The reference to new industries has been around for a long time, but the systematic conceptualization and definition of the scope of new industries in digital tourism still needs to be supplemented. Some studies have pointed out that the core of the new digital tourism industry is the innovative integration and application of digital technology with traditional tourism, resulting in a series of new tourism products, services, experiences, and scenarios [15]. Through the publication of the “Guidance Catalogue for Industrial Structure Adjustment (2024)” in December 2023, the National Development and Reform Commission made it clear that there are a total of 23 distinct types of new tourism industries. Among these, the ones that are directly associated with digital technology are “tourism information service, smart tourism, technology tourism, and other new tourism industries.”

When it comes to the tourist business, some of the new digitally associated topics include “tourism information service,” “intelligent tourism,” “technology tourism,” and “other emerging tourism mode service systems.” This paper makes reference to this directory, which is the first official document that specifies the scope of the new tourism business. The purpose of this article is to establish the new digital tourist sector. This paper describes “digital tourism as a new industry” based on current research and documents, specifically detailing how the tourism industry management body utilizes digital infrastructure, big data, the Internet of Things, artificial intelligence, blockchain, and other digital technologies to update and transform its business model, development strategy, product and service types, product forms, and integrate with the traditional tourism industry. To meet the increasing demand for digital tourism products and services, a new industry model has emerged that integrates the most effective aspects of both traditional and modern business practices in the tourism sector. Two key features of the emerging digital tourism industry stand out: first, the widespread adoption of digital technology has made it an integral part of the tourism industry’s growth, operations, and management; and second, the industry’s expansive nature allows for the constant creation of innovative digital tourism products and services.

### 2.3 Theoretical analysis and research hypotheses

Digital infrastructure development policies have a significant impact on the emergence of emerging urban tourism forms. In the urban economic system, the institutional environment is like the soil, which profoundly affects the germination and growth of the “seed” of emerging forms. According to Tan and Sheng [16], the impact of policy and market environments on urban entrepreneurial activities is particularly essential. The development, cultivation, and eventual entrepreneurial practice of new industry sectors are all dependent to a large extent on the institutional context in which new industry sectors are located. It is important to note that in China, the direction and support of policy on the promotion of urban industry is highly prominent. Industrial new-format cultivation activities are not isolated, but rather the entrepreneurial topic and the interaction of various resources and conditions in the region are [17]. Changes have been brought about in the traditional urban tourism business as a result of the policies for the development of digital infrastructure. This industry has been afflicted by major homogenization, single experiences, high risk, and other chronic difficulties. As a result of these changes, the profound incorporation of innovation into tourism products, market services, and industry demand began to increase. To start, business owners can have better insight into developing markets thanks to the digital infrastructure strategy, which encourages the use of digital technologies in the tourist industry. Thanks to advancements like 5G networks and lightning-fast broadband, entrepreneurs can now monitor the dynamics of the tourist sector in real time. Big data and AI allow them to precisely predict future demand, which in turn inspires the creation of novel goods and services like virtual reality tourism experiences and tailored smart tour guides. Secondly, digital infrastructure promotes the digital transformation of the tourism industry and improves synergy and operational efficiency. According to Xiong et al. [18], the implementation of the Internet of Things in scenic areas creates the possibility of equipment interconnection, and intelligent management systems optimize resource deployment. This results in the industry chain becoming more tightly connected, and it also reduces the operational expenses of startups. Entrepreneurs will find it easier to integrate resources and develop their entrepreneurial space as a result of the fact that each link links to the online tourist platform in real time. In addition, the strategy on digital infrastructure offers financial investments and incentives to promote new businesses that are involved in urban tourism. Government investments are the driving force behind connected businesses, the provision of technology and supporting services for startups, such as cloud computing to give arithmetic support, tax incentives and subsidies to reduce the threshold and risk of entrepreneurship, and the attraction of talent and cash. In conclusion, the implementation of digital infrastructure has the potential to enhance the whole visitor experience, boost the allure of urban tourism, and generate a higher level of market demand [19]. The development of intelligent tourism scenarios significantly enhances and simplifies the travel experience of tourists, which in turn attracts a large number of tourists, which in turn promotes the ongoing increase of the scale of the tourism market. The persistent increase in market demand creates development space for entrepreneurial firms, encourages the excitement and creativity of entrepreneurs, and motivates them to explore emerging forms, models, and service types. Moreover, the market demand continues to climb.

In summary, this paper proposes research hypothesis H1.

H1: The implementation of the Digital Infrastructure Development Policy has helped to generate emerging forms of urban tourism.

The digital infrastructure development policy has multiple mechanisms for urban tourism entrepreneurship, mainly through three core paths. First, the implementation of the policy can promote the entrepreneurial process of the new urban tourism industry by encouraging the convergence of talents. The urban tourism industry requires a comprehensive digital infrastructure to establish a high-quality development platform. This includes a fast and reliable network, sophisticated digital office equipment, and other tools that enable talent to establish a comfortable and productive work environment [20]. With the help of the digital infrastructure, experts in the tourist industry are able to obtain a more precise understanding of the demand in the market and develop tourism products and services that are more innovative and competitive. This is especially true for the big data analysis tools and virtual reality creation platforms currently available. At the same time, digital infrastructure eliminates geographical limitations, making it possible for talented individuals to participate in project collaboration and communication from a remote location, as well as expanding the opportunities for professional advancement [11]. In order to create a talent agglomeration effect, diverse talents communicate and collaborate with one another. This effect brings the collision of knowledge, experience, and innovative thinking to the entrepreneurial projects of the new tourism industry, which in turn further promotes the entrepreneurial activities of the new tourism industry in the city’s tourism sector.

Second, the implementation of digital infrastructure development policies can promote emerging forms of urban digital tourism by enhancing the level of digital technology application. The development of digital infrastructure provides a solid foundation for the wide application of digital technologies in the field of urban tourism, making the application of cloud computing, the Internet of Things, artificial intelligence, and other technologies in tourism scenarios smoother. With the help of these digital technologies, the urban tourism industry is able to realize the digital integration and management of tourism resources and the construction of an intelligent tourism service system. For example, the use of Internet of Things (IoT) technology to achieve intelligent monitoring and management of scenic area equipment, provide personalized tourism recommendations through artificial intelligence, and rely on digital technology to give rise to virtual tourism and immersive light and shadow tourism experiences and other modes. Additionally, the innovative application of digital technology provides the groundwork for the supply of new tourism products and services, which in turn dynamically meets the ever-changing needs of tourists, thereby distinguishing itself from the competition in the market and promoting the prosperity of new urban tourism entrepreneurship [21].

Last but not least, the implementation of laws for the development of digital infrastructure might help new urban tourist entrepreneurs by boosting the support they receive from the government. accompanying funding, subsidies, and incentives brought about by the digital infrastructure policy and its accompanying policies have the potential to significantly contribute to the rapid development of scenarios connected to emerging forms. In terms of policy, the government has implemented a number of preferential policies, such as tax breaks, venue subsidies, and entrepreneurial incentives, with the goal of lowering the threshold and cost of entrepreneurship and encouraging a greater number of individuals to devote themselves to urban tourism and new-format entrepreneurship [18]. In terms of funding, the government has increased its investment in digital infrastructure while setting up special support funds and guiding financial institutions to provide loan support for entrepreneurial projects to alleviate the financial pressure on startups. In addition, the government also provides technical guidance and resource docking for entrepreneurs by organizing training and building communication platforms so as to support the entrepreneurial activities of emerging urban forms in all aspects.

In summary, this paper proposes research hypothesis H2.

H2: Digital infrastructure development policy implementation influences the generation and breeding of emerging urban forms through three paths: promoting digital talent pooling, innovating digital technologies and applications, and improving government support. Spillover effects of digital infrastructure policy implementation on entrepreneurial activities in new urban tourism sectors. The resources and technologies involved in digital infrastructure, such as network communication and data processing, have the characteristic of transcending geographical boundaries and can be rapidly disseminated and shared in a wide range. However, when it comes to the actual impact on new tourism entrepreneurial activities in different regions, it will have different effects due to the differences in regional spatial relations, which are manifested in the promotion of neighboring regions and the siphoning effect on non-neighboring regions.

For neighboring regions, the implementation of digital infrastructure policies will have a significant catalytic effect. First, geographic proximity makes information dissemination more convenient and rapid. The information bridge built by digital infrastructure allows information such as tourism market dynamics, changes in consumer preferences, and the application of emerging tourism technologies to circulate quickly among neighboring regions [22]. By combining the qualities of local tourism resources, entrepreneurs in neighboring regions can seize market opportunities quickly, gain knowledge from advanced experiences, and create innovative products and services. This, in turn, spurs more entrepreneurial projects in new tourism industries. Second, the development of digital infrastructure has greatly improved the communication, logistics, and transportation systems in the region [23]. This improvement helps to actualize the sharing of resources and complementary advantages, encourages synergistic development, strengthens economic ties between regions, and sets the groundwork for the establishment of the cross-regional tourism industry chain. As an example, adjacent areas can collaborate to develop cross-regional tourism routes and advertise each other’s brands to increase visitor numbers. This kind of collaboration promotes entrepreneurial activity by making the market more favourable to new types of entrepreneurship. But digital infrastructure plans might have a draining effect on areas that aren’t next to one another. The development of digital infrastructure will lead to a rapid increase in the competitiveness of the tourism industry in core cities or regions, attracting a large concentration of high-quality resources such as capital, talent, and technology. Non-contiguous regions are at a disadvantage in the competition for resources due to their distance. On the one hand, funds are more inclined to be invested in areas with perfect digital infrastructure and strong development prospects, leading to the plight of new tourism industry entrepreneurship in non-contiguous areas facing a shortage of funds [24]. On the other hand, high-quality tourism professionals and innovative talents will also be attracted by the numerous development opportunities and favorable conditions in the core regions, resulting in a serious brain drain and weakened entrepreneurial and innovative capabilities in non-adjacent regions [4]. In addition, from the perspective of market competition, regions with advanced digital infrastructure are able to create more high-quality and diversified tourism products and service systems by relying on cutting-edge technologies and efficient operation modes. This makes these regions much more attractive to tourists, thus further capturing the tourism market share of non-neighboring regions. These circumstances have significantly suppressed the entrepreneurial activities of new settlers in non-neighboring regions.

In summary, this paper proposes research hypothesis H3.

H3: While the implementation of digital infrastructure development policies generates spillover effects to neighboring regions, it also creates siphon effects in non-neighboring regions.

## 3. Research design

### 3.1 Selection and description of variables

#### 3.1.1 Explained variables.

Drawing on the methodology of Zhao and Lin [25], this study employed Python tools to conduct systematic data collection and processing for the explained variable. First, based on the conceptual definition of emerging digital tourism forms, seven core keywords—namely “immersive tourism,” “technology-enabled tourism,” “digital-intelligent tourism,” “digital tourism”, “virtual tourism”, “metaverse tourism”, and “smart tourism”—were selected as core filtering indicators. In the second step, we utilized the Selenium library to mimic browser behavior and called up the public search interfaces of two popular platforms in China for retrieving business data: Tianyancha and Qichacha. We used the research period to set our screening criteria, and we crawled in batches the basic information of newly registered enterprises in Chinese urban areas from 2012 to 2024. This included the number of enterprises, enterprise names, unified social credit codes, registered addresses, registered capital, and establishment dates. We used the Pandas package to save the information as structured data. The third step was to delete duplicate enterprise information using unified social credit codes. We also selected an acceptable range for registered capital to eliminate aberrant amounts. Lastly, we filtered out samples with registered addresses that could not be matched to specific cities. Finally, Stata 20.0 was used to conduct Principal Component Analysis (PCA) on the enterprise information corresponding to the seven keywords. After passing the adaptability verification test, the PCA with a cumulative variance contribution rate exceeding 90% were selected for weighted calculation of the comprehensive index. Ultimately, the annual comprehensive level of emerging forms of urban digital tourism for each city was synthesized to quantify the development level of emerging tourism forms in different cities.

#### 3.1.2 Core explanatory variables.

In this paper, the dummy variable of Broadband China policy pilot cities, which is set as the core explanatory variable, consists of the interaction term between treatment group and treatment year. The list of Broadband China pilot cities published by the Ministry of Industry and Information Technology for three consecutive years from 2014–2016 creates an ideal quasi-natural experimental environment for this paper. Therefore, this paper uses the list of pilot cities to develop the core explanatory variables. This is done by assigning a value of 1 to the variable whether it is a digital infrastructure pilot city if a city has been identified as a Broadband China pilot city in the observation year; conversely, a value of 0 is assigned if it has not been classified as a pilot city.

#### 3.1.3 Control variables.

In order to deeply analyze the net promotion effect of digital infrastructure construction on regional tourism emerging forms entrepreneurial activities, this paper refers to the research ideas of Bai et al. [17] and Jiao et al. [20], and combines with the actual situation of urban entrepreneurship, the following control variables are selected: (1) the level of economic development. As an important cornerstone of regional entrepreneurial activities, the regional economic development status is an important influence on the entrepreneurial activities of emerging urban forms. This paper adopts the logarithmic value of regional GDP to measure. (2) Financial development level. Financial development plays an active role in stimulating the entrepreneurial vitality of urban tourism. This paper utilizes the ratio of the year-end loan balance of financial institutions to the gross regional product for measurement. (3) Industrial structure. The proportion of the tertiary industry in the city positively influences entrepreneurship in the tourism industry. The tertiary industry is defined in this research by its value added ratio relative to the gross regional product. (4) Degree of education. An increase in the city’s educational attainment can foster an entrepreneurial spirit that can lead to the creation of novel tourist attractions. In this study, we utilize the enrollment rate in public secondary schools as a percentage of the total population as our unit of analysis. (5) The amount consumed. Emerging kinds of tourism and entrepreneurship are both boosted by an increase in local consumption. As a measure of consumption, this research looks at the regional GDP as a percentage of total consumer goods sales. Service level for communication (6). In order to foster and grow entrepreneurial endeavors in urban tourism, the quality of communication service—an essential component of contemporary society—is of paramount importance. This paper uses the number of mobile phone users as a metric for this. seventhly, the network supporting postal and telecommunication services. The growth of postal and telecommunication services is conducive to increasing the connectivity of innovative resources in the region, promoting the dissemination and diffusion of knowledge within the region, and facilitating the entrepreneurship of emerging forms, which is measured by the logarithmic value of the total amount of postal and telecommunication services in this paper.

#### 3.1.4 Mechanism variables.

Increasing government support, boosting technical innovation and application, and improving the level of talent agglomeration are the three ways in which the digital infrastructure policy, as stated earlier, can influence the entrepreneurial activities of urban digital tourism. This study presents three mechanism variables—the intensity of government support, the amount of technical innovation and application, and the level of talent agglomeration—to conduct a thorough analysis of the intrinsic influence mechanism. The following is a measurement of the mechanism variables based on typical academic procedures, such as Pan and Xu [26]: (1) the talent agglomeration level, which is a measure of the concentration of talent in a city as a whole, as measured by the proportion of the population employed by computer-related businesses. The higher the percentage, the greater the concentration of computer-related skills in the city, a phenomenon known as talent agglomeration. (2) The quantity of utility model patents filed in the city within a given year is a good indicator of the degree of technological advancement and implementation. A city’s level of technological innovation and application can be characterized by its utility model patent count, which provides an intuitive presentation of the city’s active degree of technological invention and the research and efforts of new technologies in practical application. (1) The ratio of a city’s spending on science and technology this year to its spending on general public expenditure is a measure of the intensity of government support. The larger the ratio is, the more resources the government has invested in science and technology, which demonstrates the government’s support for the related fields.

### 3.2 Modeling

#### 3.2.1 Baseline model.

To estimate the impact of the pilot policy of Broadband China on the entrepreneurial activities of the new tourism industry in the region, this paper adopts a multi-period DID model for regression analysis, and the specific measurement model is as follows:


$$NTE_{it}=\beta_{\text{0}}+\beta_{\text{1}}(Treat\times Time{)}_{it}+\lambda Z_{it}+\mu_{t}+\nu_{i}+\varepsilon_{it}\;$$ (1)

In Eq.(1), *i*(*i* = 1,2,...,287) is the city, *t*(*t* = 2012,2013,...,2024) is the year, and the explanatory variable *NTE*_*it*_ denotes the level of development of new tourism entrepreneurial activities in city *i* in year *t.* $(Treat\times Time{)}_{it}$is the DID estimator, if city *i* is the pilot area of Broadband China policy, Treat is 1, otherwise it is 0. Time indicates the pilot time of city *i,* if city *i* is set up as the pilot area of Broadband China in year *t*, Time is 1, and 0 otherwise. $Z_{it}$is other influential factors that may affect the entrepreneurial activities. $\mu_{t}$denotes the time fixed effect. $\nu_{i}$is the individual fixed effect. $\varepsilon_{it}$ denotes the randomized perturbation term.

#### 3.2.2 Mechanism testing model.

After verifying the impact of the pilot policy of Broadband China, this paper builds upon the ideas of Jiang Ting [27] and establishes the following model based on the benchmark regression model to verify the mechanism of digital infrastructure influencing the emerging forms of urban digital tourism entrepreneurship:


$$M_{it}=\alpha_{\text{0}}+\alpha_{\text{1}}(Treat\times Time{)}_{it}+\lambda Z_{it}+\mu_{t}+\nu_{i}+\varepsilon_{it}$$ (2)

$M_{it}$in Eq.(2) denotes the three mechanism variables proposed by the above theoretical analysis: talent agglomeration level, the level of technological innovation application, and government support intensity. The rest of the symbols are consistent with Eq.(1). Given the significant effect of the core explanatory variables in the benchmark regression, we analyze the coefficient of the pilot policy $(Treat\times Time{)}_{it}$on the mechanism variable $M_{it}$. If $M_{it}$is significant, it indicates the existence of the mechanism path.

#### 3.2.3 Spatial effects test model.

To further test the spatial spillover effects, the following model is set up:


$$\begin{array}{c} NTE_{it}=\beta_{\text{0}}+\sum {\mathrm{\backslash nolimits}}_{s=\text{5}\text{0}}^{\text{4}\text{0}\text{0}}\;\beta_{\text{1}}N_{it}^{s}+\lambda Z_{it}+\mu_{t}+\nu_{i}+\varepsilon_{it} \end{array}$$ (3)

Building on the benchmark regression, Eq.(3) excludes samples from the original pilot cities to examine the net effect in neighboring cities. Following the approach of Cao [28], introduces the explanatory variable $N_{it}^{s}$, the parameter s represents the geographical distance between cities (in kilometers, with s ≥ 50), calculated using the spherical distance based on latitude and longitude coordinates. Specifically, if in year *t*, there exists a Broadband China pilot city within the spatial range of (s – 50, s] kilometers from city *i*, then $N_{it}^{s}$ takes the value of 1; otherwise is 0. For example, $N_{it}^{s}$ is used to determine whether a Broadband China pilot city was present within 50 kilometers of city *i* in year *t*. The regression coefficient of $N_{it}^{s}$ measures the impact of the Broadband China pilot policy on emerging tourism forms in nearby cities. Furthermore, this paper reports regression results at 50-kilometer intervals (i.e., s = 50, 100,..., 350, 400) to examine the spatial spillover effects of the policy. By comparing the statistical significance of regression coefficients across different distance thresholds, we assess how the policy’s influence varies with spatial proximity.

### 3.3 Data sources and descriptive statistics

This paper explores the impact of digital infrastructure development policies on entrepreneurial activities in new tourism sectors in urban areas using city-level panel data from 2012 to 2024. In view of the availability of data and the changes to administrative divisions in the time dimension, some cities with serious missing data are excluded from this paper. We eventually generated a balanced panel data set that includes 297 Chinese cities at the prefecture level after screening, and you can see the descriptive statistics for it in Table 1. The following sources provide the empirical data: As a first step, we use a Python crawler software to collect data about entrepreneurial activities in prefecture-level cities from the websites of Tianyancha and Qichacha. Two of China’s most prominent business data platforms are Tianyancha and Qichacha. Their information comes from government databases like China Judgments Online and the National Enterprise Credit Information Publicity System. Both systems have seen heavy application in empirical studies conducted in the fields of economics and management, and the results are known to be thorough and dependable. In addition, the MIIT-published pilot city documents are the source for the list of cities that have adopted the Broadband China pilot program. Third, the control variables (economic, financial, education, consumption, industrial, postal infrastructure, and communication levels) are derived from various sources (e.g., the EPS data platform, the China Urban Construction Statistical Yearbook, and the China Urban Statistical Yearbook 2013–2022). When values are missing, they are reasonably filled in using linear interpolation.

**Table 1:** Definition of variables and descriptive statistics.

Variables name	Definition	Average value	Standard deviation
Emerging forms of urban digital tourism	Comprehensive level of emerging forms of urban digital tourism calculated based on PCA	0.001	1.2218
Broadband china pilot city	Dummy variable	0.2826	0.4503
Level of economic development	Gross urban product	16.6913	1.0054
Level of financial development	Ratio of the balance of loans from financial institutions to GDP at the end of the year	1.1719	0.6921
Consumption level	Ratio of total retail sales of consumer goods to regional GDP	0.3728	0.1148
Industrial structure	Ratio of tertiary sector value added to GDP	43.8119	10.1978
Educational level	Ratio of the number of students enrolled in general secondary schools to the household population	0.0510	0.0429
Post and telecommunication infrastructure	Logarithmic value of total post and telecommunication	11.1666	3.5340
Level of communication services	Cell phone subscribers at the end of the year	498.9965	542.5731
Talent agglomeration level	Ratio of urban computer workers to employees	0.5291	0.9376
Level of technological innovation and application	Number of utility model patent applications filed by standardized cities in the year	0.0188	0.0657
Government support intensity	Ratio of urban science and technology expenditures to local general public budget expenditures for the year	0.0170	0.1760

## 4. Analysis of results

### 4.1 Baseline regression results

This paper adopts a progressive regression strategy to conduct the study. In Table 2, columns (1) and (2) present the regression results without the inclusion of control variables; column (3) shows the regression without the addition of time and individual fixed effects; and column (4) shows the regression results with the addition of control variables, time, and individual variables. According to these findings, the impact of the Broadband China pilot policy on emerging form cultivation coefficients are consistently positive, even when controlling for time fixed effects and other variables. This suggests that building digital infrastructure plays a significant role in encouraging the growth of new tourist entrepreneurial activities. The results show that the installation of digital infrastructure clearly promotes the growth of new entrepreneurial activities in urban tourism, proving hypothesis H1.

**Table 2:** Benchmark regression results.

Variables	Emerging forms of urban digital tourism			
	(1)	(2)	(3)	(4)
Broadband China pilot	0.5847*** (8.0870)	0.3096*** (4.2665)	0.2709*** (4.1174)	0.3014*** (4.5454)
Level of economic development			0.2903*** (4.4648)	0.4015*** (5.3172)
Level of financial development			0.1880*** (4.0094)	0.2219*** (3.6896)
Consumption level			−0.5843*** (−3.5226)	−0.6010*** (−3.4926)
Industrial structure			−0.0050** (−2.2655)	−0.0017 (−0.6010)
Educational level			−0.0997 (−0.4700)	−0.0794 (−0.3812)
Post and telecommunication infrastructure			−0.0200*** (−3.3262)	−0.0193*** (−3.1050)
Level of communication services			0.0019*** (4.9055)	0.0020*** (4.9674)
Constant term	−0.1652*** (−6.7183)	−0.0875*** (−3.4827)	−5.4344*** (−5.7343)	−7.5011*** (−6.1556)
Time fixed effect	No	Yes	No	Yes
Individual fixed effect	Yes	Yes	No	Yes
Observed value	3861	3861	3861	3861
Adjustment r^2^	0.563	0.584	0.617	0.617

Note: Corresponding t-values are in parentheses, and *, **, and *** indicate results at the 10%, 5%, and 1% significance levels, respectively. “Yes” means the control variable has been added to the regression model, or fixed effects have been included; “No” denotes that the control variable has not been added or that fixed effects have not been incorporated.And the same applied below.

### 4.2 Robustness tests

#### 4.2.1 Parallel trend test and placebo test.

**Fig 1:**
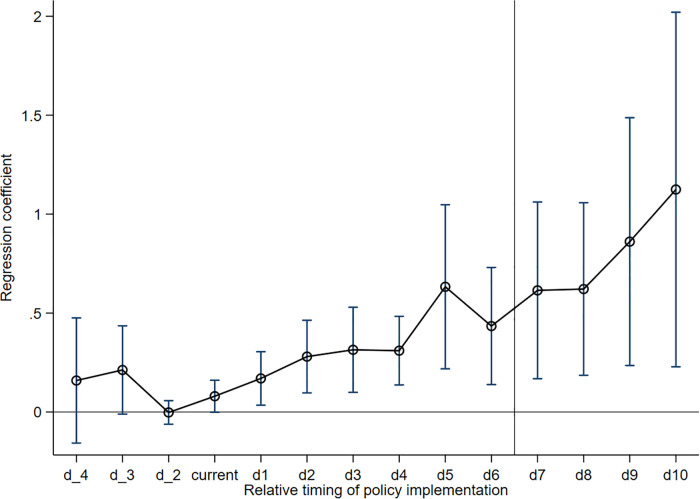
Parallel trend test. Note: d_i denotes year i before policy implementation, and d_i denotes year i after policy implementation.

When utilizing the DID model for policy assessment, the treatment and control groups need to satisfy a key precondition, i.e., a parallel trend before the policy implementation. To effectively avoid the interference of the multicollinearity problem, the previous period before the implementation of the policy is excluded from the sample in this paper, which is used as the base period. The estimation results of the parallel trend test are presented in Fig 1, from which it can be seen that the estimated coefficients of the core explanatory variables do not show significance (with confidence intervals covering 0) in the periods before the official implementation of the Broadband China pilot strategy. This result is a strong indication that before the implementation of the policy, there was no significant difference between pilot and non-pilot cities in terms of entrepreneurial activities in the new tourism sector. The implementation of the policy gradually highlighted its promotion effect on the cultivation of emerging forms in cities.

**Fig 2:**
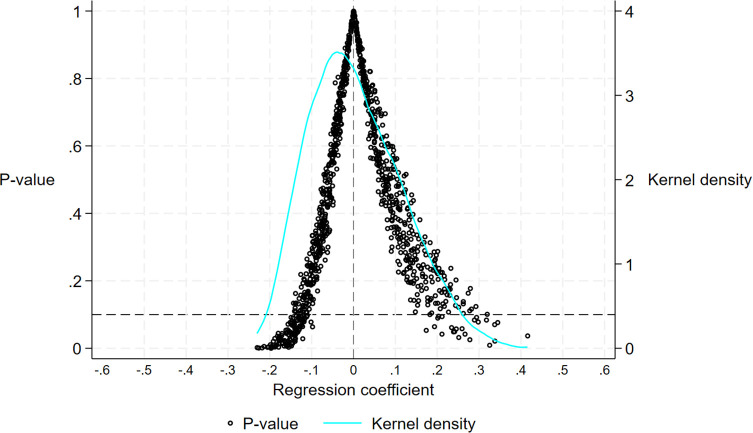
Placebo test.

To guarantee the reliability of the results of the regression analysis and exclude the interference of random factors, this paper adopts the strategy of randomly designating the control group and the experimental group and implements 1,000 repetitions of sampling as a means of constructing a placebo test. Specifically, within the full sample, randomly select cities with the same number of pilot cities, set them as the “pseudo-experimental group,” and identify the remaining cities as the “pseudo-control group.” We used a multi-period DID model to perform regression analysis after randomly assigning the year of policy implementation to each of these “pseudo-experimental group” locales. Fig 2 shows the distribution of p-values and regression coefficients for 1000 random samples. The dots representing p-values and kernel density estimation curves indicate that the regression coefficients follow a nearly normal distribution. The regression coefficients of the Broadband China pilot policy in the randomized experiments cluster around 0, which differs significantly from the true estimates. As a result, the estimation results are robust and unaffected by the omitted variables, supporting hypothesis H1. Additionally, the P-values are mostly greater than 0.1, further confirming this.

#### 4.2.2 Considering the impact of other policies.

In addition to the Broadband China pilot policy, the implementation of other policies may also have an impact on the cultivation of new tourism entrepreneurial activities. The pilot policy of the national-level big data comprehensive experimental zone will promote the construction of urban information infrastructure, thereby stimulating regional innovation capacity and fostering new tourism entrepreneurial activities. This paper sets up the pilot policy variable of the big data comprehensive pilot zone; if the city belongs to the pilot policy list of the big data comprehensive pilot zone in that year and later, the variable is assigned to 1, and the rest is assigned to 0. The pilot list originates from the official websites of the government and various functional departments. Second, the opening of high-speed rail signifies the facilitation of urban transportation conditions, which contributes to the development of the local tourism industry and empowers the entrepreneurial activities of the new tourism industry. This paper establishes a dummy variable to represent the opening of high-speed rail, using the year when local high-speed rail lines open as the starting point. The variable is assigned a value of 1 for the year of opening and subsequent years, while it is assigned a value of 0 for all other years. Lists of high-speed rail opening times are compiled from announcements made by the railroad department. Third, the telecom universal service pilot policy for the purpose of broadening network coverage also contributes to the construction of information infrastructure in prefectural-level cities to stimulate regional innovation capacity, which may have an impact on the conclusions of this paper, and therefore, a dummy variable for telecom universal service is set, which is assigned a value of 1 if the city belongs to the pilot list in the current year and thereafter, or 0 otherwise, and the list of the pilot cities is obtained from the website of the Ministry of Industry and Information Technology. According to the fourth point, the national tourism standardization demonstration units that were announced by the Ministry of Culture and Tourism contribute to the leadership of the industry’s development, optimize the tourism environment, enhance the level of urban tourism management and service quality, and have a positive impact on the activities of entrepreneurs. The purpose of this article is to establish a dummy variable for the national tourist standardized demonstration units. If the city is included on the list both in the current year and in subsequent years, then the variable is assigned a value of 1, and if it is not included, then it is assigned a value of 0. The General Office of the Ministry of Culture and Tourism is the source of the list of communities. The fifth point is that the development of innovative cities has a positive impact on the innovative behavior of regional small and medium-sized businesses. These businesses work toward the establishment of a technological innovation system that will facilitate the deep integration of industry, academia, and research. Additionally, these businesses work to accelerate the transfer and transformation of major scientific and technological achievements, which in turn helps to cultivate the entrepreneurial activities of tourism in new industries. Both the Ministry of Science and Technology and the National Development and Reform Commission are responsible for compiling the list of cities that are currently undergoing construction.

This paper incorporates five variables into the regression model, to exclude the impact of other policies on the entrepreneurial activities of emerging forms. These variables are the pilot policy of a national-level big data pilot zone, the opening of high-speed rail, the pilot project of universal telecommunication service, the national tourism standardization demonstration policy, and the policy of innovative city construction. The specific results are shown in Table 3, and the paper is based on the policies that have been discussed above. The findings demonstrate a thorough consideration of the effects of various policies. The results show that, after considering the effects of other policies, the regression coefficient of the Broadband China pilot policy is still significantly positive, indicating that it has a significant role in promoting the cultivation of new tourism entrepreneurial activities in the city, which further confirms the validity of hypothesis H1.

**Table 3:** Robustness tests excluding other policy effects.

Variables	Emerging forms of urban digital tourism				
	(1)	(2)	(3)	(4)	(5)
Broadband China pilot	0.3003***	0.3065***	0.3098***	0.3188***	0.2031***
	(4.5575)	(4.6808)	(4.7352)	(4.7925)	(3.1702)
Big data comprehensive pilot zone policy	0.2478** (2.5539)	0.2462** (2.5584)	0.2432** (2.5372)	0.2436** (2.5446)	0.2300** (2.5001)
High-speed rail opened		−0.2576*** (−6.0977)	−0.2459*** (−5.8601)	−0.2407*** (−5.7530)	−0.1879*** (−4.5562)
Telecom universal service pilot policy			−0.2020*** (−5.5201)	−0.1946*** (−5.3699)	−0.1162*** (−3.5287)
National tourism standardization demonstration units				−0.1481*** (−3.4583)	−0.1647*** (−3.7813)
Innovative cities construction					0.6980*** (8.6807)
Constant term	−7.7621*** (−6.2083)	−7.6345*** (−6.2149)	−7.6461*** (−6.2352)	−7.5883*** (−6.2055)	−6.6115*** (−6.0859)
Control variable	Yes	Yes	Yes	Yes	Yes
Time fixed effect	Yes	Yes	Yes	Yes	Yes
Individual fixed effect	Yes	Yes	Yes	Yes	Yes
Observed value	3861	3861	3861	3861	3861
Adjustment r^2^	0.619	0.622	0.623	0.623	0.637

#### 4.2.3 Substitution of explanatory variables.

The benchmark regression uses PCA to measure the explanatory variables; however, this method may not be able to visualize the changes in the number of enterprises in the new tourism entrepreneurial activities in the city, and it does not take into account the differences in the registered capital of the enterprises and other circumstances. Therefore, this paper employs two methods to re-evaluate the new tourism entrepreneurial activities and perform regression analysis.

Based on the above seven keywords, this paper simply aggregates the number of newly registered enterprises in the same city each year as an explanatory variable so as to intuitively reflect the development of the entrepreneurial activities of the new tourism industry in each city.

The specific operation process is as follows: firstly, calculate the average registered capital for each city and each enterprise corresponding to each keyword; then, calculate the ratio of the average registered capital of each enterprise belonging to each keyword in each city to the sum of the average registered capital of all keywords; lastly, use the ratio as the weight to summarize the number of newly registered tourism enterprises under each keyword in that year, and use the summarized result as an explanatory variable. The summarized results are used as the explanatory variables. The relevant results are presented in columns (1) and (2) of Table 4. The table shows that the Broadband China pilot policy’s regression coefficient remains significantly positive even after replacing the explanatory variables.

**Table 4:** Other robustness tests.

Variables	Simple addition	Weighted summary	Exclusion of special cities	One-period lagged explanatory variables
	(1)	(2)	(3)	(4)
Broadband China Pilot	3.5890*** (3.6149)	0.5648*** (4.9276)	0.1361*** (2.6056)	0.2042*** (2.7492)
Constant term	−38.4414** (−2.0037)	−13.0689*** (−6.1437)	−4.3568*** (−4.8755)	−8.0021*** (−4.9313)
Control variable	YES	YES	YES	YES
Time fixed effect	YES	YES	YES	YES
Individual fixed effect	YES	YES	YES	YES
Observed value	3861	3861	3458	3564
Adjustment of R^2^	0.409	0.666	0.629	0.590

#### 4.2.4 Consideration of the impact of special areas.

Considering that municipalities and provincial capitals occupy a special position in China’s urban system, to make the results more generalizable, the four municipalities and the provincial capitals of each province are excluded from the regression analysis in this paper, and the regression operation is repeated. Table 4’s third column presents the findings of the regression analysis. After eliminating these particular cities, the findings suggest that the regression coefficients of the key explanatory variables align with the baseline regression coefficients, indicating that they are moving in the same direction. Furthermore, this result provides additional confirmation of the robustness of the results of the benchmark regression, which in turn strengthens the reliability of the conclusions drawn from the study.

#### 4.2.5 One-period lagged explanatory variables.

There may be a time lag in the cultivation of new tourism entrepreneurial activities, and in order to gain insights into the possible lagged effects of policy impacts, this paper re-regresses the explanatory variables with one period of lagging, and the results are shown in Column (4) of Table 4. The results show that the regression coefficients of the Broadband China pilot on the entrepreneurial activities of emerging forms are significantly positive, indicating that the pilot policy can still significantly promote the generation of emerging forms after lagging the explanatory variables by one period, which supports Hypothesis H1.

#### 4.2.6 Tests based on the PSM-DID.

In the selection of pilot cities for Broadband China, there is a self-selection bias that tends to select cities with relatively complete digital infrastructure and a high proportion of digitized production technology applications. And such cities usually have conditions conducive to the development of new tourism entrepreneurial activities, which makes the treatment and control groups in the original model systematically different in nature. Given this information, the purpose of this research is to reevaluate the impact that the Broadband China policy has had on the implementation of the policy by applying the means of Propensity Score Matching-Difference in Differences (PSM-DID). To be more specific, this paper adheres to the concept of cross-section matching, treats all control variables as covariates, matches the samples using 1:1 nearest neighbor matching, caliper matching, and radius matching, and then then performs regression analysis on the samples that have been matched once more. The specific results are presented in Table 5. According to the findings presented in Table 5, the regression coefficients are consistent with the results of the baseline regression under a variety of matching approaches.

**Table 5:** PSM-DID matching results.

Variables	Nearest neighbor matching	Caliper matching	Radius matching
	(1)	(2)	(3)
Broadband China pilot	0.4057** (2.3981)	0.2692** (2.2344)	0.2307*** (2.7542)
Constant term	−9.1243*** (−3.6625)	−10.6953*** (−3.4409)	−5.5608*** (−2.8674)
Control variable	YES	YES	YES
Time fixed effect	YES	YES	YES
Individual fixed effect	YES	YES	YES
Observed value	1396	2019	3788
Adjustment R^2^	0.172	0.181	0.168

#### 4.2.7 Exclusion of endogeneity.

A preliminary comparative analysis of digital infrastructure conditions in pilot and non-pilot cities reveals that pilot cities are generally better than non-pilot cities in terms of digital infrastructure, with obvious natural differences, which suggests that the selection of pilot cities may not be completely random, and that there is a self-selection bias that causes endogenous problems in the model, leading to a biased assessment of policy effects. This suggests that the selection of pilot cities may not be completely random, and there is self-selection bias, which causes endogenous problems in the model, leading to bias in the evaluation of policy effects. Therefore, this paper adopts the Heckman two-step method to deal with the above endogeneity problem. In the first stage, this paper constructs the interaction term between terrain relief and regional GDP per capita as well as the control variables as covariates, and uses the Probit model to calculate the Inverse Mills Ratio (IMR). In the second stage, in order to solve the endogeneity dilemma that may be caused by policy choices, this paper incorporates the IMR into the benchmark regression model for regression analysis. The results show that the regression coefficients corresponding to the Broadband China pilot policy are significantly positive (see Table 6). This finding provides strong support for hypothesis H1 and fully confirms the rationality of the hypothesis.

**Table 6:** Heckman two-stage regression results.

Variables	First stage	Second phase
	Indicator variable	Explanatory variable
Explanatory variable	–	0.4967*** (2.7237)
	–	
IMR	–	4.9287* (1.7803)
	–	
IV	0.0519*** (3.4954)	–
		–
Constant term	−9.1218*** (−13.9450)	−31.2240** (−2.1004)
Control variable	YES	YES
Time fixed effect	–	YES
Individual fixed effect	–	YES
Observed value	3,858	3,858

### 4.3 Mechanism analysis

Table 7 shows the test results of the mechanism of the digital infrastructure policy to promote the development of emerging forms of urban digital tourism, according to which it can be seen that hypothesis 2 is valid. Specifically analyzed as follows:First, the talent agglomeration level. In the development of emerging forms of urban digital tourism, talent is a key element. The test results of the talent pooling mechanism are displayed in column (2). Due to the fact that the regression coefficients of the core explanatory variables are significantly positive, it is evident that the digital infrastructure policy has the potential to effectively drive the convergence of computer talents and other information talents in order to better serve the emerging forms of urban digital tourism. The second factor is the degree of technological innovation and technology use. The advancement of technology is a significant factor that plays a driving role in the promotion of the development of new types of urban digital tourism. Column three illustrates the influence that the policy on digital infrastructure has on technical innovation. The coefficient of this policy is significantly positive at the 1% level, which indicates that it is able to successfully motivate the city to plan innovation and new applications. This brings us to the third effect: the strength of government backing. According to the findings presented in Column 4, the coefficient of digital infrastructure policy on government support is both positive and significant. This suggests that digital infrastructure policy has the potential to encourage the local government to increase the amount of money it spends on science and technology. Furthermore, this financial support effect can assist the city in developing its tourism industry sector.

**Table 7:** Mechanism analysis.

Variables	Emerging forms of urban digital tourism	Talent agglomeration level	Level of technological innovation and application	Government support Intensity
	(1)	(2)	(3)	(4)
Broadband China Pilot	0.3014*** (4.5454)	0.0739* (1.8835)	0.0124*** (4.8472)	0.0017** (2.5198)
Constant term	−7.5011*** (−6.1556)	1.4629 (1.1946)	−0.1998*** (−3.5048)	−0.2289*** (−12.4984)
Control variable	YES	YES	YES	YES
Time fixed effect	YES	YES	YES	YES
Individual fixed effect	YES	YES	YES	YES
Observed value	3861	3835	3243	2934
Adjustment R^2^	0.617	0.677	0.864	0.789

### 4.4 Heterogeneity analysis

#### 4.4.1 Tourism endowment conditions.

The development and improvement of digital infrastructure can significantly improve the dissemination speed of regional tourism information and the ability of tourists to access information [18]. However, digital infrastructure is a “lone tree,” and the natural differences in the endowment of regional tourism resources may affect the implementation of digital infrastructure policies. The richness and level of regional tourism resources will directly affect the regional layout of the new tourism industry. Specifically, cities with higher endowments of tourism resources are more likely to form industrial cluster effects and promote the cultivation and development of new tourism industries and new modes. In view of this, this paper counts the number of 4A and 5A scenic spots in each prefecture-level city from 2012 to 2024 in accordance with the current guidelines for the classification of tourist attraction quality levels. And take the median as the boundary, divide the cities into two groups of non-tourism endowment and tourism endowment, and then carry out regression analysis, and the results are presented in Table 8. As can be seen from Table 8, the regression coefficient of the tourism-endowed cities is 0.2783, which is valid at a significant level of 5% and is higher than that of the non-tourism-endowed cities. This implies that the promotion effect of digital infrastructure represented by the Broadband China pilot policy on the cultivation of emerging forms is more significant in cities with high tourism resource endowment.

**Table 8:** Heterogeneity analysis.

Variables	Emerging forms of urban digital tourism					
	Non-tourism endowment city	Tourism endowment city	Ordinary city	Core city	Non-historical Famous city	Historical famous city
Broadband China Pilot	0.2096*** (3.8765)	0.2783** (2.1359)	0.1210** (2.2853)	0.5353* (1.7142)	0.2920*** (3.2521)	0.3279** (2.3197)
Constant term	−3.0227*** (−4.0217)	−19.6746*** (−5.4096)	−3.9874*** (−4.5428)	−38.1692*** (−4.6989)	−6.5105*** (−4.3377)	−7.5834*** (−3.2820)
Control variable	YES	YES	YES	YES	YES	YES
Time fixed effect	YES	YES	YES	YES	YES	YES
Individual fixed effect	YES	YES	YES	YES	YES	YES
Observed value	1966	1880	3406	455	2731	1113
Adjustment R^2^	0.529	0.622	0.630	0.607	0.631	0.608

#### 4.4.2 Urban organizational capacity.

Within the framework of China’s administrative management system, the different administrative levels of cities will lead to a significant differentiation of urban grassroots organizational capacity [29], which will have an impact on the effectiveness of the Broadband China pilot policy. Generally speaking, the higher the administrative level of a city, the stronger its resource deployment capacity and grassroots organizational capacity. In view of this, this paper classifies municipalities, provincial capitals, and sub-provincial cities as core cities and other cities as ordinary cities and carries out regression by groups, and the specific regression results are presented in Table 8. The results of the regression are shown in Table 8. The analysis shows that the Broadband China pilot policy has promoted the cultivation of emerging forms in both ordinary cities and core cities. However, compared with the core cities, the marginal utility of the policy implementation in ordinary cities is lower. This may be due to the fact that core cities, as the strategic core of economic development in each region, have more prerequisites for the popularization of digital infrastructure, and their grassroots organization and resource allocation capabilities are better than those of ordinary cities [21], which makes the difference in the administrative hierarchies of cities have an impact on the effect of the Broadband China pilot policy. This makes the difference in the administrative hierarchy of cities have an impact on the effect of the Broadband China pilot policy.

#### 4.4.3 National historical and cultural cities.

The historical and cultural status of different cities may affect the cultivation process of the new tourism industry [30]. As historical and cultural cities often have a deep cultural heritage, their cultural relics and natural landscapes have a natural advantage for the development of resource-dependent tourism, and digital technology-enabled tourism has a significant additive effect. In order to group and analyze the other cities, this paper uses the list of national historical and cultural cities as a criterion to divide the first three batches of cities into a historical group and the rest into non-historical groups. Table 8 displays the results, which demonstrate that the pilot program is effective in promoting both historic and non-historic cities. However, the policy’s marginal effect is more noticeable in the category of national historic cities. This is largely due to the fact that, as part of its promotion of Broadband China digital infrastructure, historic cities are displaying and experiencing natural tourism products in new ways thanks to the deep integration of digital technology and high-quality resources. The main reason for this is that historic cities, which are supported by the policy of Broadband China’s digital infrastructure, have embraced digital technology and high-quality resources. This has allowed them to display and experience natural tourism products in a much larger way, while also providing tourists with immersive tourism experiences. In comparison to non-historic cities, these cities are better suited to fostering the development of new forms.

### 4.5 Analysis of spatial effects

**Fig 3:**
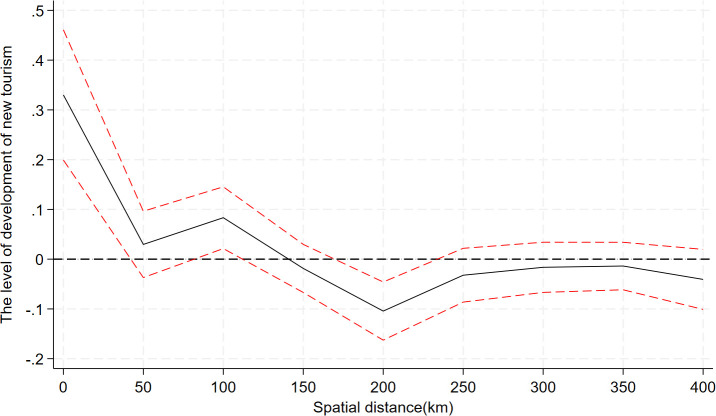
Spatial effects.

In order to investigate whether there is a spillover effect of the Broadband China policy pilot at the geospatial level, based on the theoretical analysis in the previous section, the constructed spatial spillover model is used to investigate the spatial effect of the development of digital infrastructure on the cultivation of new tourism industries in neighboring cities. Fig 3 demonstrates the trend of explanatory variables with spatial distance (confidence interval is 95%). Specifically, the driving effect of the Broadband China pilot on the development of emerging forms in neighboring cities shows an inverted S-shaped change with increasing spatial distance, which first decreases, then increases, and then decreases. In the range of 50–100 km around the pilot city, digital infrastructure has a significant pulling effect on the development of tourism, and the positive effect of the pilot on the cultivation of the new tourism industry in the neighboring cities is weakening after exceeding 100 km; while in the range of 150–200 km, the siphoning effect is obvious, which is not conducive to the cultivation of tourism in the cities within the range. This suggests that the digital infrastructure promoted by the Broadband China policy pilot is closely related to spatial distance and that there are obvious heterogeneous spatial effects of digital infrastructure on the cultivation of emerging forms in neighboring cities.

The reason for this is that the catalytic effect of the Broadband China policy pilots on emerging forms is concentrated in the 50–100 km range. When the spherical distance between neighboring cities and the pilot city is too close, the good level of digital infrastructure in the pilot city forms a clustering shadow area. As a result, tourism resources of neighboring cities gradually converge to the pilot city due to the scale effect of the agglomeration area. The Broadband China policy pilot will only benefit the local digital tourism industry when the spherical distance is 50–100 kilometers, free from the agglomeration shadow area. If the pilot city is too far away, its positive impacts diminish and become a siphon effect. This may be because spatial remoteness raises information dissemination and factor flow costs. Information dissemination and production factor allocation are crucial to new tourist industry development [31]. If neighboring cities are too far from pilot cities, the information dissemination advantage of digital infrastructure is gradually weakened in long-distance transmission, and it is difficult for neighboring cities to quickly obtain the advanced experience and innovative mode of digital tourism development in pilot cities, so they cannot quickly integrate into the trend. On the other hand, the pilot cities occupy a more favorable competitive position in the tourism market by virtue of their digital infrastructure advantages, and their perfect digital infrastructure platforms attract more tourist flows and form a strong market agglomeration. The gap between the pilot cities and the remote neighboring cities in terms of digital infrastructure capacity leads to an imbalance in digital tourism resources. At the same time, the convenient payment and intelligent marketing services brought by digital infrastructure give the pilot cities a greater advantage in tourism consumption experience, further attracting tourists and related tourism enterprises to gather, resulting in the continuous flow of production factors such as tourism resources, sources of tourists, and industrial investment from neighboring cities to the pilot cities, which is not conducive to the cultivation and development of new tourism industries in remote neighboring cities.

## 5. Conclusions and discussion

### 5.1 Conclusions

The Chinese government’s sustained support for digitalization policies has created transformative opportunities for urban tourism development. This study employs the Broadband China pilot policy as a quasi-natural experiment, utilizing a multi-period DID approach to investigate how digital infrastructure policies drive entrepreneurship in urban digital tourism sectors. Four important findings emerge from the investigation. First, there is strong evidence from studies of parallel trends, placebo effects, variable substitutions, and endogeneity controls that shows how developing digital infrastructure greatly encourages new types of urban digital tourism. Secondly, the strategy is able to accomplish its goals by means of three complementary processes: increasing the concentration of digital talent, encouraging technical innovation, and strengthening government support structures. Third, cities with unique cultural capital, efficient deployment of administrative resources, and an abundance of tourist resources see a multiplicative effect of policy efficacy. As a fourth point, the results of the spatial analysis show a “proximity paradox”: nearby cities, like the Rhine-Ruhr regional cluster in Germany, reap the benefits of industrial synergies and information sharing, while faraway cities, like the Amazon in Brazil, risk marginalization as a result of resource drainage effects.

### 5.2 Talking about it

The study’s findings shed light on the interdependence of digital infrastructure and new types of urban digital tourism in China, and they also serve as a resource for the international advancement of digitalization in the tourism industry, thanks to their fundamental reasoning and real-world consequences. Digital infrastructure serves as the technical backbone for new business models in tourism, with its core essence lying in lowering the technological barriers for market players and optimizing resource allocation efficiency to foster innovative tourism scenarios. This underlying logic holds true across diverse national contexts. Generally, against the backdrop of steadily rising economic development levels and household consumption capabilities, government-led tourism infrastructure policies often catalyze robust growth in regional tourism industries—a pattern validated globally. For instance: In 2019, Germany launched the Innovative Capability Enhancement in Tourism initiative, supporting Lübeck Bay’s development of an “intelligent crowd control” traffic management system. Under this policy framework, novel formats like virtual tours and online performances flourished across the country’s urban attractions. In 2023, Singapore’s Tourism Board and Infocomm Media Development Authority jointly introduced the Industry Digital Plan (IDP) for tourism attractions. With state backing, the IDP effectively integrates emerging technologies to advance the digital transformation of physical sites and empowers operators to adopt digital solutions. The EU’s European Capital of Smart Tourism 2022 award, meanwhile, spurred the adoption of smart tourism practices by promoting accessibility, sustainability, and digitalization, accelerating sectoral growth across Europe. Despite variations in technological starting points and policy instruments, these cases collectively underscore the research insight that synergies between digital infrastructure and policy frameworks drive the emergence of new tourism models. Such experiences offer valuable pathways for other nations seeking to harness digital infrastructure for tourism industry advancement.

Based on the aforementioned findings and discussions, this paper derives the following policy implications:

First, it is essential to expand the coverage of digital infrastructure policies and deepen their impact on the upgrading of the digital tourism industry in urban areas. From the perspective of urban regions themselves, proactive efforts should be made to enhance the construction, supporting facilities, and utilization of digital infrastructure, thereby seizing the developmental opportunities it presents. This includes accelerating the adoption of emerging digital tourism forms and services, such as intelligent tourism navigation, virtual reality tourism, and immersive experiences, to facilitate the transformation of urban tourism toward greater intelligence and convenience.

Second, considering the heterogeneous endowments of different cities, policies should be implemented in a categorized and phased manner to achieve “localized adaptation.” Specifically, cities abundant in tourism resources should tailor policy implementation plans based on their comparative advantages, fostering a stronger awareness of integrating digital technologies with the tourism industry during policy execution. Priority should be given to promoting the digital transformation of the tourism sector while strengthening urban organizational capacity to accelerate tourism-oriented development through administrative measures. For cities with rich historical and cultural heritage, efforts should focus on deepening the integration of digital technologies with high-quality cultural resources, expanding the presentation and experiential dimensions of historical and cultural assets, and enabling immersive tourism experiences to revitalize and effectively preserve these resources. For instance, augmented reality (AR) projects could be developed in historical cities to restore ancient sites, allowing tourists to experience time travel and immersive participation. Cities reliant on natural resources could establish digital twin scenic areas, integrating meteorological and ecological monitoring and tourist behavior data to shift tourism from “sightseeing-based” to “deeply participatory.” Metropolitan leisure cities could explore “metaverse + consumption” models to avoid homogeneous competition.

Finally, priority should be given to encouraging and nurturing tourism cities with exemplary policy implementation outcomes, leveraging their demonstration and spillover effects to promote inter-city collaborative tourism development. Examples include establishing cross-city tourism alliances and encouraging core cities to export smart scenic area management systems to less-developed cities, with the latter reciprocating through ecological resource sharing, thereby forming a “technology-for-resources” complementary model.

The positive effects of China’s digital infrastructure policies clearly demonstrate that accelerating digital infrastructure development is an effective pathway for driving the digital transformation of the tourism industry and fostering new tourism business models. This provides valuable insights for the development of urban tourism and its digitalization on a global scale. As substantiated by the case studies examined in this paper—Germany’s Innovation Demonstration Projects for Enhancing Tourism Competitiveness, Singapore’s Tourism (Attractions) Industry Digital Plan (IDP), and the EU’s European Capital of Smart Tourism 2022—accumulated empirical evidence conclusively demonstrates that digitalization has become an indispensable component of future urban development, and “digital solutions” are equally essential for the advancement of urban tourism industries.
